# Anti-cancer effects of Ajwa dates (*Phoenix dactylifera L.)* in diethylnitrosamine induced hepatocellular carcinoma in Wistar rats

**DOI:** 10.1186/s12906-017-1926-6

**Published:** 2017-08-22

**Authors:** Fazal Khan, Tariq Jamal Khan, Gauthaman Kalamegam, Peter Natesan Pushparaj, Adeel Chaudhary, Adel Abuzenadah, Taha Kumosani, Elie Barbour, Mohammed Al-Qahtani

**Affiliations:** 1 Biochemistry Department, Faculty of Science, King Abdulaziz University, Jeddah, Saudi Arabia; 2 Center of Excellence in Genomic Medicine Research (CEGMR), King Abdulaziz University (KAU), PO BOX 80216, Jeddah, 21589 Kingdom of Saudi Arabia; 3 Center of Innovation in Personalized Medicine, King Abdulaziz University, Jeddah, Saudi Arabia; 4 King Fahd Medical Research Center, King Abdulaziz University, Jeddah, Saudi Arabia; 5 Department of Agriculture, Faculty of Agricultural and Food Sciences, American University of Beirut (AUB), Beirut, Lebanon

**Keywords:** Ajwa date extract, Hepatocellular carcinoma, In vivo, Anti-oxidants, Cytokines, Quantitative real-time PCR

## Abstract

**Background:**

Hepatocellular carcinoma (HCC) accounts for major cancer-related deaths despite current advanced therapies. Treatment and prognosis of HCC is better in patients with preserved liver function. Many natural products including ajwa dates (*Phoenix dactylifera L.*), are claimed to have hepatoprotective and HCC inhibitory effects, but most lack scientific validation. To prove our hypothesis, we attempted to evaluate the HCC inhibitory effects, and other beneficial properties of the aqueous extract of ajwa dates (ADE) in a rat model of diethylnitrosamine (DEN) induced liver cancer.

**Methods:**

Thirty-two male rats were divided into four groups of eight each as follows, Group A: untreated control; Group B: DEN control (180 mg/kg bw), Group C: DEN + ADE 0.5 g/kg bw; and Group D: DEN +1.0 g/kg bw. Rats from all groups were assessed for liver cancer progression or inhibition by evaluating histological, biochemical, antioxidant enzyme status, cytokines and gene expression profiles.

**Results:**

DEN treatment Groups (B, C, D) showed histological features of HCC and in rats treated with ADE (Groups C, D) partial to complete reversal of normal liver architecture was observed. Antioxidant enzymes such as superoxide dismutase (SOD), glutathione reductase (GR), glutatione peroxidase (GPx) and catalase (CAT) were increased, while the liver enzymes alanine aminotransferase (ALT), aspartate aminotransferase (AST) and alkaline phosphatase (ALP) levels and lipid peroxidation were significantly decreased in Group C and Group D compared to Group B. Pro-inflammatory cytokines such as interleukin (IL)-1α, IL-1β,, GM-CSF) were increased in the serum of rats in Group B while the anti-tumor cytokines (IL-2, IL-12) were increased in ADE treated Groups (C, D). In addition, Alpha-Feto Protein (AFP) and IL-6 gene expression levels were upregulated in Group B, while they were significantly downregulated in ADE treated Groups (C, D).

**Conclusions:**

ADE helped in the reversal of DEN damaged liver towards normal. Restoration of anti-oxidant enzymes, liver enzymes, cytokines balance and gene expression to normal levels following ADE treatment indicates that ADE improves liver function and inhibits HCC. ADE can, therefore, be used together with conventional therapeutics for HCC.

## Background

Hepatocellular carcinoma (HCC) is one of the leading cause of cancer-associated deaths, and globally it is three times more common in men than women [1]. HCC is one of the leading cause of cancer related deaths in the Kingdom of Saudi Arabia (KSA). HCC is the 4^th^ most common cancer in males and 8^th^common cancer in females accounting for 4.3% of the reported cancer cases in the year 2013 [2]. A global estimation of 40,710 new cases and 28,920 deaths due to liver and intrahepatic bile duct cancers in 2017 highlights the need for improvement in current management strategies [1]. Common risk factors for liver cancer include heavy alcohol consumption, hepatitis B virus (HBV), hepatitis C virus, obesity, tobacco smoking, diabetes and genetic factors. Partial hepatectomy, embolization and sorafenib (Nexavar^®^) [3] offer partial to complete success depending upon the stage of the disease.

The incidence of high mortality and associated side effects following chemotherapy and/or radiotherapy increase the demand for alternative medicine for the cancer treatment. Not surprisingly, many potent anticancer compounds were isolated from the plants viz. doxorubicin, taxol, etoposide, cisplatin, vinblastine, vindesine, vincristine and topotecan [4]. Given the current interests in alternative medicine, it is imperative that much-concerted effects are undertaken to identify novel compounds that will aid therapy and also help to avoid its unjustifiable use.

Date fruit (*Phoenix dactylifera L.*) is a native fruit of arid region, largely cultivated as an economical and food crop in the Middle East, Southern Europe, North Africa, South America, India, and Pakistan [5]. Of the many varieties of date fruits, ajwa dates are unique for its medicinal properties and these are specifically grown in the city of Al-Madina Al-Munawwarah, KSA [5]. Ajwa dates are a rich source of energy, and readily provide sugars, proteins, vitamins, high dietary fibers, minerals and fats [6]. They contain various phytochemicals like sterols, polyphenols, flavonoids and glycosides [6]. Ajwa dates have hypolipidemic, antioxidant, anti-inflammatory, cardioprotective, nephroprotective and hepatoprotective effects [7]. In addition, the concentrated polyphenols and ex vivo digested extracts from ajwa dates inhibited growth and proliferation of colon cancer Caco-2 cell line [8]. Also, (1 → 3)-β-D-glucan, which is an essential component of dates had an inhibitory effect on sarcoma-180 in vivo in mice [9]. Recently, we identified that methanolic extract of ajwa dates inhibited human breast adenocarcinoma (MCF-7) by causing cell cycle inhibition and induction of apoptosis [10]. Interestingly, so far no studies have been done to identify the effects of ajwa or other dates on hepatic cancer.

Many in vitro and in vivo models of hepatic cancer have been utilized in the past to evaluate various drugs. In vivo hepatic cancer model development is done using various chemical methods, of which diethylnitrosamine (DEN) is a chemical carcinogen, commonly utilized to induce liver carcinogenesis by increasing oxidative stress and liver damage [11]. Hence in the present study, we used the DEN-induced hepatic cancer model in Wistar rats and evaluated the cancer inhibition effects of the aqueous extract of ajwa dates (ADE). Histological, biochemical, antioxidant enzyme status, as well as the related cytokines and gene expression profiles was studied.

## Methods

### Animals

The animals used in this study was approved by the Institutional Ethics Committee, King Abdulaziz University (KAU), Saudi Arabia vide approval number HA-02-J-008. Thirty-two albino male rats (Wistar strain, 5-6 weeks old) that weighed between 100 g - 120 g were purchased through the animal holding unit (AHU), King Fahd Medical Research Center (KFMRC, KAU). All rats were housed in the AHU under standard animal housing conditions of 25 °C ± 3 °C, 55 ± 2% humidity, 12 hrs day/night cycle, and had access to standard rat pellet and water ad libitum.

### Preparation of aqueous extract of ajwa date (ADE)

Ajwa dates used in the present study were sourced from Al Madina Dates Co. (Tomoor), Madinah Al Munawarah, Kingdom of Saudi Arabia. Ajwa dates sample was deposited with the Biology Department, Faculty of Science, KAU. Dr. Dhafer Ahmed Al-Zahrani, Plant Taxonomist confirmed the genus, species and origin of date fruit (Specimen voucher number: *P. dactylifera L*. #PD17569). Ajwa dates flesh was mixed with ultrapure distilled water at a ratio of 1:3. The date suspension was continuously agitated and incubated at 25 °C on a shaker (at 60 rpm) placed within the incubator (25 °C) for 48 hrs. The date-water extract was then filtered through muslin cloth and Whatman no. 1 filter paper. The filtrate was then centrifuged at 4500 rpm for 10 min. The supernatant was again filtered and the aqueous extract of ajwa dates (ADE) thus obtained was stored at −80 °C until use.

### Experimental design

Wistar male rats (*n* = 32) were acclimatized for 1 week prior to start of experiments. Rats were randomly divided into four experimental groups (*n* = 8). HCC induction was done using diethylnitrosamine (DEN). Briefly, DEN was dissolved in corn oil and two doses (180 mg/kg body weight (bw); at 15 days interval) were administered orally to all rats in Group B, C and D using intragastric gavage. Untreated control (Group A) was administered vehicle (corn oil) alone. Treatment with ADE was started on the next day following DEN treatment. All rats in Group C and Group D were treated with ADE 0.5 g/kg bw and 1.0 g/kg bw respectively, daily for 10 weeks; while the rats in Group A (untreated control) and Group B (DEN control) were administered vehicle alone. Following completion of treatment, the animals were sacrificed using an injection of chloral hydrate (400 mg/kg bw; i.p). Blood and liver tissues were collected from all the animals in different experimental groups. Serum was separated by centrifugation (2500 rpm × 10 min) and stored at −80 °C until further analyzed. Part of the liver tissue was fixed in 10% neutral buffered formalin for 24 h or in RNA later for subsequent use in histological and molecular analysis respectively.

### Histology

Formalin fixed liver tissues were dehydrated using gradient concentrations of ethanol; then washed in xylene and embedded in paraffin wax. Tissue blocks were sectioned at 5-6 μM thickness, deparaffinized and stained with hematoxylin and eosin and analyzed under microscope (Olympus Instruments, Tokyo, Japan).

### Anti-oxidant enzymes

The following anti-oxidant enzymes, namely superoxide dismutase (SOD, Sigma 19,160), glutathione reductase (GR, Sigma GRSA), glutathione peroxidase (GPx, Sigma CGP1) and catalase (CAT, Sigma CAT100) were analyzed using respective kits and manufacturer’s protocols. SOD and CAT were end point assays and the absorbance was obtained at 440 nm and 520 nm respectively. GR and GPx were kinetic assays and the absorbance at 412 nm and 340 nm was obtained every 10 s respectively.

### Lipid peroxidation (LPO) assay

LPO assay was carried out using the Sigma kit (MAK085) according to the manufacturer’s instructions. Following end point assay, the absorbance at 532 nm was determined spectrophotometrically.

### Liver enzymes

The activity of the following liver enzymes namely, alanine aminotransferase (ALT, Abcam 105,134), aspartate aminotransferase (AST, Abcam 105,135) and alkaline phosphatase **(**ALP, Abcam 83,369) were measured using respective kits according to the manufacturer’s instructions. Following incubation for 5 min, the absorbance at 570 nm (ALT, AST) and 405 nm were determined spectrophotometrically.

All spectrophotometric analysis were done using the microplate reader (SpectraMax® i3x, Molecular Devices, Sunnyvale, CA).

### Multiplex cytokine assay

Serum samples from ADE treated and control rats were analyzed for both pro-inflammatory and anti-inflammatory cytokines using the Rat Cytokine Magnetic 10-Plex Panel (Novex^®^) according to the manufacturer’s instructions. Briefly, the magnetic beads in solution were added and washed twice with 1X wash buffer. Standards (1:3 serial dilution) and samples (1:2 dilution) were prepared and added to the washed beads and incubated on an orbital shaker at 500 rpm for 2 hrs. The plate was then incubated with detection antibodies for 1 hr and streptavidin-RPE antibodies for 30 min incubation. Between incubations with different antibodies, the plate was washed twice with wash buffer. Following antibody incubation, the plate was finally washed thrice and resuspended in wash buffer and analyzed using on MAGPIX^®^ instrument (Luminex, USA). Data obtained was analyzed using the Luminex^®^ xPONENT^®^ multiplex assay analysis software.

### Quantitative real time-PCR (qRT-PCR)

Total ribonucleic acid (RNA) was extracted from the liver tissue of both control and treated rats using Pure Link® RNA Mini Kit (Ambion™, Thermo Fischer Scientific) according to the manufacturer’s protocol. On column, DNase-I treatment was included in the protocol. First-strand cDNA synthesis was carried out using random hexamers (High Capacity cDNA Reverse Transcription Kit, Applied Biosystems). Primers were designed using qPrimerDepot (National Institute of Heath, USA) and PrimerBank (The Massachusetts General Hospital, USA), and the primer sequences are given in Table 1. The qRT-PCR analysis was performed using the ABI StepOnePlus™ Real-Time PCR System (Applied Biosystems) using SYBR green dye master mix and relative quantification was performed using the comparative 2 ^–ΔΔCt^ method which analyzes the relative change in gene expression based on Ct values (fluorescence denoting cDNA copy numbers) of housekeeping gene and gene of interest).

**Table 1:** Primer sequences

Genes	Primer type	Sequence
*AFP*	Forward	5′-CTGTATGCTCCCACCATTCTTT-3′
	Reverse	5′-TTGATGCTCTCTTTGTCTGGAA-3′
*IL6*	Forward	5′-CCGGAGAGGAGACTTCACAG-3′
	Reverse	5′-CAGAATTGCCATTGCACAAC-3′
*GAPDH*	Forward	5’GACTCTACCCACGGCAAGTT-3′
	Reverse	5’GGTGATGGGTTTCCCGTTGA-3′

### Statistical analysis

Statistical analyses were performed using Prism GraphPad version 6.0. One way ANOVA (analysis of variance) and student unpaired t-test for comparisons between groups and within groups respectively. All the values were expressed as mean ± SEM (standard error of the mean). Asterisk (*) and hash (#) indicates statistical significance, with the level significance for comparisons set at *P* < 0.05.

## Results

### Histology

**Fig. 1:**
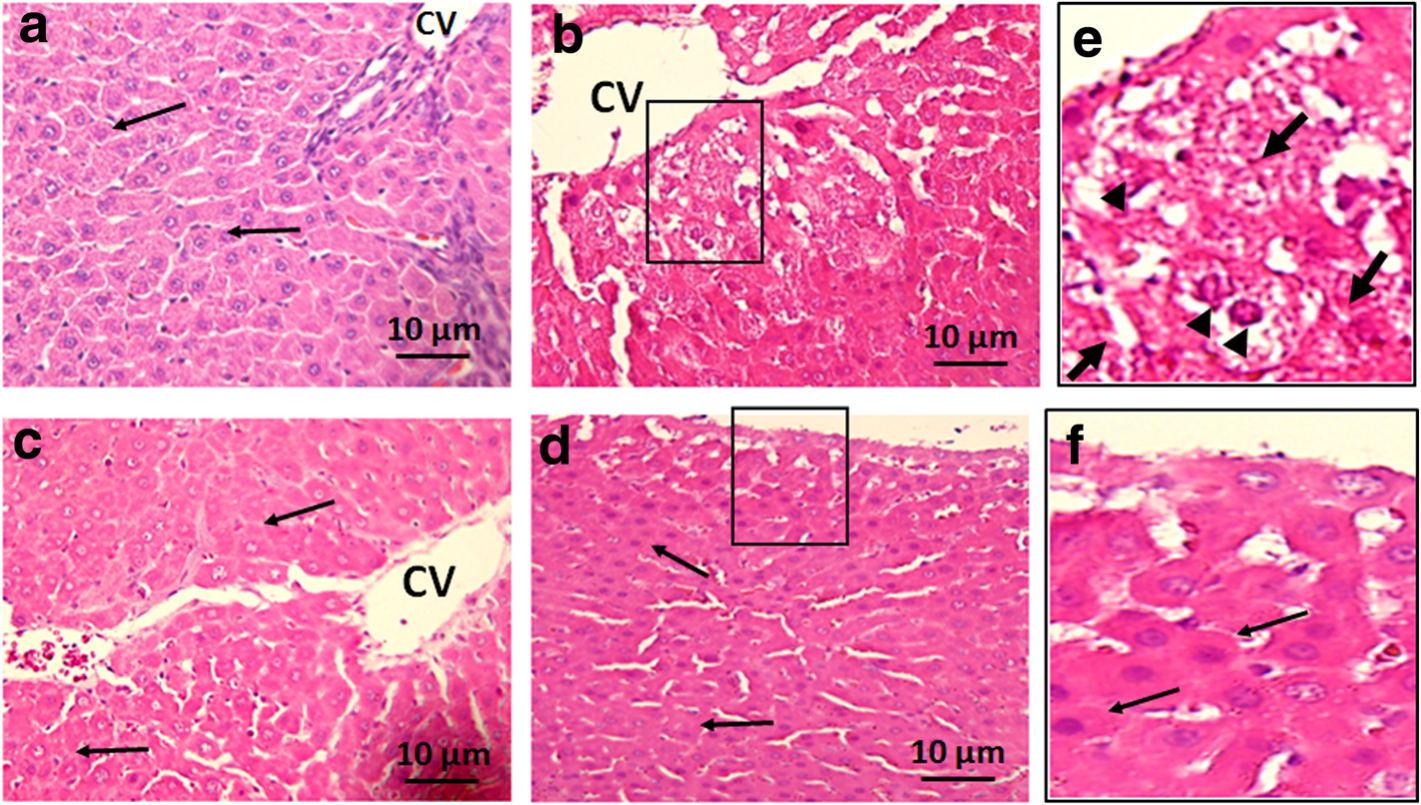
Haematoxylin and eosin (H&E) staining: Representative images of the liver sections (**a**, **b**, **c**, **d**) from rats treated with aqueous date extract (ADE) following DEN induced HCC are shown above. **a** - untreated control (Group A) shows normal hepatocytes, intact cell membrane and central vain; **b** - DEN control (Group B) liver shows altered hepatocyte morphology, with loss of cell membrane, increased nuclear size and connective tissue infiltration; **c**, **d** - the ajwa treated Groups (C: 0.5 g/kg bw; D: 1.0 g/kg bw) shows normal liver pattern with radially arranged hepatocytes similar to that of the control and this was more pronounced in Group D than Group C. Magnification (40X). Inset (**e**) is the magnified image of the boxed area in B (DEN treated), where changes in cell morphology, nuclear pattern and connective tissue infiltration are clearly demonstrated. Inset (**f**) is the magnified image of the boxed area in D (Ajwa 1 g/kg bw), which shows normal histological pattern of the liver. Thin black arrows indicate normal hepatocyte morphology. Thick black arrows indicate connective tissue infiltration. Black arrow heads indicate abnormal nuclear morphology

Liver sections from the control group showed normal lobular pattern with central vein and peripheral portal veins (Fig. 1a). In contrast, the animals treated with DEN showed evidence of inflammatory cell infiltration and fibrosis. The normal lobular pattern was replaced with clusters of atypical cells; hepatocytes with increased nuclear to cytoplasmic ratio and dense connective tissue (Fig. 1b). However, treatment with ADE (0.5 g/kg bw and 1.0 g/kg bw) promoted partial to complete reversal of liver architecture (Fig. 1c and d).

### Antioxidant enzymes

**Fig. 2:**
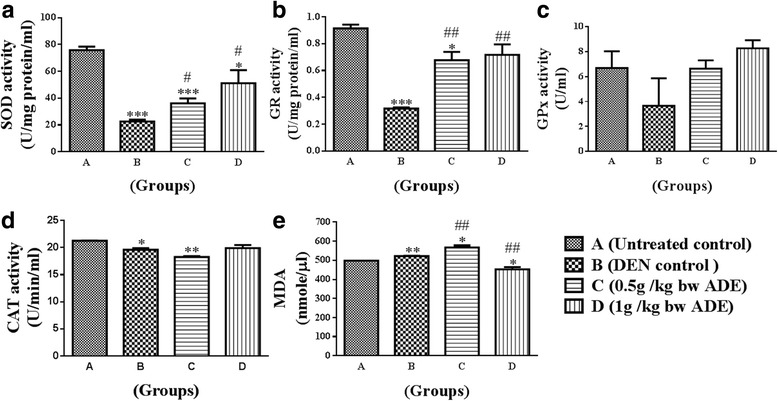
Analysis of antioxidant enzymes and lipid peroxidation levels from the serum samples of rats treated with aqueous date extract (ADE) following DEN induced HCC. Antioxidant enzymes namely, (**a**) - superoxide dismutase (SOD); (**b**) - glutathione reductase (GR); (**c**) - glutathione peroxidase (GPx); (**d**) - catalase (CAT); and (**e**) lipid peroxidase levels were analyzed. One-way ANOVA and student t test were used to compute statistics between groups and within groups respectively. All values were expressed as mean ± SEM. Comparisons were made between untreated control (Group A) and DEN control (Group B) and their significance were represented using asterisk (*p < 0.05 and **p < 0.01, ***p < 0.001) and hash (# *p* < 0.05, ## *p* < 0.01 and ### *p* < 0.001) respectively

In general, the activity of the anti-oxidant enzymes SOD, GR, GPx and CAT were decreased in treated Groups (B, C, D) compared to normal untreated control (Group A). Compared to DEN control, the ADE treated Groups (C, D) demonstrated increases in activity for SOD, GR and GPx and these increases in values were statistically significant (*p* < 0.05) (Fig. 2a–c). However, CAT demonstrated an increase only in Group D and this was not statistically significant (Fig. 2d).

### Lipid peroxidation (LPO) assay

Malondialdehyde (MDA) levels as determined by LPO assay, showed mild increases in Groups (B, C), and a decrease in Group D compared to untreated normal control (Group A). Compared to Group B (DEN control), LPO demonstrated an increase in Group C and a decrease in Group D these values in the ADE treated groups were statistically significant (*p* < 0.05) (Fig. 2e).

### Liver enzymes

**Fig. 3:**
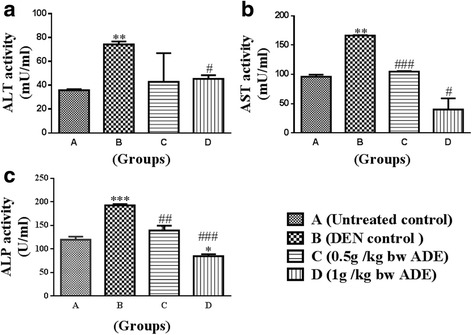
Analysis of liver enzymes from the serum samples of rats treated with aqueous date extract (ADE) following DEN induced HCC. Liver enzymes namely, (**a**) alanine aminotransferase; (**b**) aspartate aminotransferase and (**c**) alkaline phosphatase were analyzed. One-way ANOVA and student t test were used to compute statistics between groups and within groups respectively. All values were expressed as mean ± SEM. Comparisons were made between untreated control (Group A) and DEN control (Group B) and their significance were represented using asterisk (*p < 0.05 and **p < 0.01, ***p < 0.001) and hash (# *p* < 0.05, ## *p* < 0.01 and ### *p* < 0.001) respectively

In general, the activity of the liver enzymes ALT, AST and ALP were increased in treated Groups (B, C, D) compared to normal untreated control (Group A). Compared to DEN control, the ADE treated Groups (C, D) demonstrated decreases in activity for ALT, AST and ALP and these decreases in values statistically significant (*p* < 0.05) (Fig. 3a–c).

Additional results from anti-oxidant enzymes (SOD, GR, GPx, CAT), LPO and liver enzymes (ALT, AST, ALP) are summarized in Table 2.

**Table 2:**
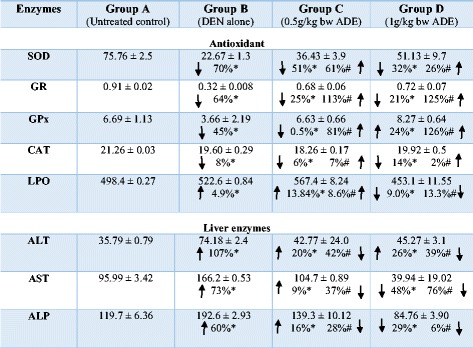
Summary of results of liver and antioxidant enzymes

*Percentages compared to Group A; # percentages compared to Group B

### Cytokines

In general, the cytokines analyzed demonstrated a decrease in the proinflammatory cytokines (IL-1β, IL-1α, and GM-CSF) and an increase in the anti-tumor cytokines IL-2 and IL-12 compared to Group B (DEN control).

**Fig. 4:**
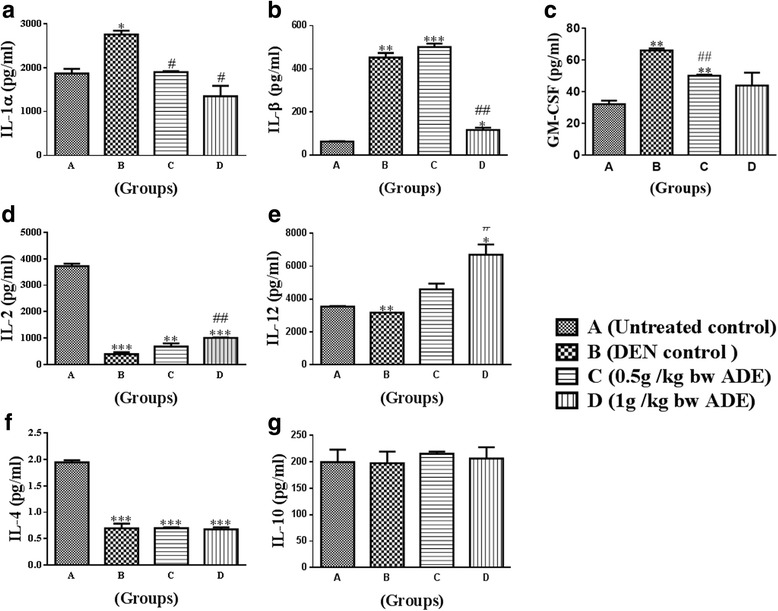
Analysis of serum cytokines from the serum samples of rats treated with aqueous date extract (ADE) following DEN induced HCC. The following cytokines namely, (**a**) IL-1α; (**b**) IL-1β, (**c**) GM-CSF, Th1 [(**d**) IL-2; (**e**) IL-12] and Th2 [(**f**) IL-4; (**g**) IL-10] were analyzed. One-way ANOVA and student t test were used to compute statistics between groups and within groups respectively. All values were expressed as mean ± SEM. Comparisons were made between untreated control (Group A) and DEN control (Group B) and their significance were represented using asterisk (*p < 0.05 and **p < 0.01, ***p < 0.001) and hash (# *p* < 0.05, ## *p* < 0.01 and ### *p* < 0.001) respectively

The concentrations of IL-1α were 1867.3 4 ± 84.78, 2754.82 ± 69.12, 1899.23 ± 17.07 and 1349.25 ± 192.88 for the Groups A, B, C and D respectively. Only the increase in Group B (by 48%) statistically significant (*P* < 0.05), compared to Group A (Fig. 4a). Compared to Group B, IL-1α was decreased in Group C by 31% and Group D by 51%, and these mean decreases in values in the ADE treated groups were statistically significant (*p* < 0.05) (Fig. 4a).

The concentrations of IL-1β were 63.27 ± 0.86, 452.7 ± 15.05, 501.74 ± 11.45 and 116.78 ± 7.28 for the Groups A, B, C and D respectively. IL-1β concentrations were increased in the treated Groups (B, C, D by 614%, 691%, 83% respectively) compared to Group A (Fig. 4b). Compared to Group B, IL-1β increased moderately in Group C (by 10%) and decreased in Group D (by 74%). All mean increases or decreases in values were statistically significant (*p* < 0.05) (Fig. 4b).

The concentrations of GM-CSF were 32.27 ± 2.17, 66.12 ± 10.01, 50.16 ± 0.65 and 43.99 ± 8.13 for the Groups A, B, C and D respectively. GM-CSF concentrations significantly increased (*P* < 0.05) in treated Groups (B, C, by 106%, 56.25% respectively) compared to Group A (Fig. 4c). Compared to Group B, only the decrease in Group C by 24% was statistically significant (*p* < 0.05) (Fig. 4c).

The concentrations of IL-2 were 3724 ± 95.97, 389.8 ± 76.57, 677.6 ± 119.6 and 1008 ± 6.16 for the Groups A, B, C and D respectively. IL-2 concentrations were significantly decreased (*P* < 0.05) in treated Groups (B, C, D by 90%, 82%, 73%) compared to Group A (Fig. 4d).

Compared to Group B, IL-2 demonstrated increases in Group C by 74% and Group D by 159%; however, only the increase observed in Group D was statistically significant (*p* < 0.05) (Fig. 4d).

The concentrations of IL-12 were 3548 ± 30.25, 3177 ± 1.79, 4604 ± 346.86 and 6703 ± 628.5 for the Groups A, B, C and D respectively. IL-12 concentration decreased in Group B (by 4%), increased in Group D (by 89%) compared to Group A; and increased in Group D (by 111%) compared to Group B. These values were statistically significant (*p* < 0.05) (Fig. 4e).

The concentrations of IL-4 were 1.946 ± 0.03, 0.6958 ± 0.08, 0.6991 ± 0.01 and 0.6764 ± 0.03 for the Groups A, B, C and D respectively. IL-4 concentrations were statistically decreased (*P* < 0.05) in treated Groups (B, C, D by 64.3%, 64%, 65.3%) compared to Group A (Fig. 4f). Compared to Group B the observed increase in Group C (by 0.6%) and decrease in Group D (by 3%) were not statistically significant (Fig. 4f).

The concentrations of IL-10 were 199.6 ± 23.77, 197.6 ± 2.18, 215.3 ± 4.19 and 206.3 ± 21.41 for the Groups A, B, C and D respectively. IL-10 concentration was decreased in Group B (by by 1.5%), while this was increased in Groups (C, D by 7.5%, 3.0% respectively) compared to Group A. Compared Group B, the IL-10 demonstrated increases in Group C (by 9.0%) and Group D (by 4.6%) and all these increases or decreases in values were not statistically significant (*p* < 0.05) (Fig. 4g).

### Quantitative real time PCR (qRT-PCR)

**Fig. 5:**
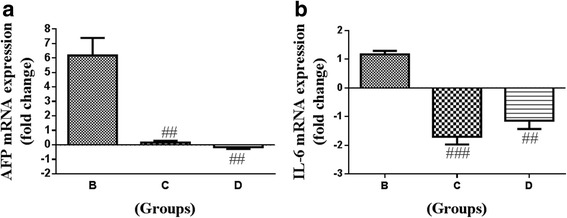
Real time gene expression analysis of the liver tissue from rats treated with aqueous date extract (ADE) following DEN induced HCC. (**a**) AFP; (**b**) One-way ANOVA and student t test were used to compute statistics between groups and within groups respectively. All values were expressed as mean ± SEM. Comparisons were made between untreated control (Group A) and DEN control (Group B) and their significance were represented using asterisk (*p < 0.05 and **p < 0.01, ***p < 0.001) and hash (# *p* < 0.05, ## *p* < 0.01 and ### *p* < 0.001) respectively

Gene expression demonstrated biphasic responses in treated Groups (B, C, D) compared to Group A. Alfa-fetoprotein (AFP) gene expression was upregulated by 6-fold and 0.61-fold in Group B and Group C respectively, while this was downregulated by 0.15-fold in Group D, compared to Group A (Fig. 5a and b). Interleukin 6 (IL-6) gene expression was upregulated by 2-fold in Group B, while this was downregulated by 1.7-fold and 1.2-fold in Group C and Group D respectively compared to Group A. Compared to Group B the decreases observed in both AFP and IL-6 were statistically significant (*P* < 0.05).

## Discussion

**Fig. 6:**
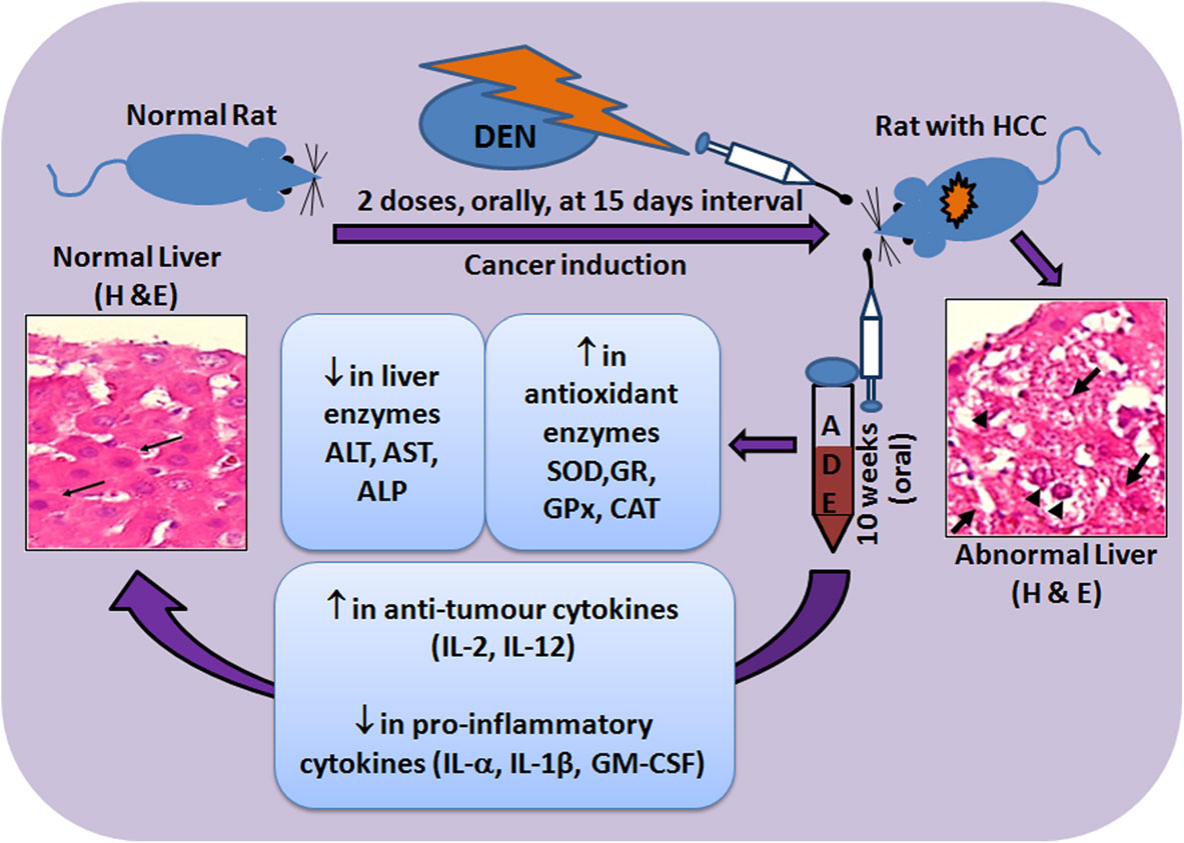
Summary of the effect of ADE on the DEN-induced liver cancer in Wistar rats

Increased incidence of HCC in the west are partly due to an earlier epidemic of HCV, prevalence of obesity and diabetes which are associated with non-alcoholic fatty liver disease (NAFLD) whereas it is declining in the Eastern population especially in Taiwan and Singapore in response of active implementation of HBV immunization, and the prognosis remains poor with an overall survival rate below 9% [12, 13]. HCC does not respond to chemotherapy and fairly responds to radiotherapy and therefore have limited therapeutic options when the tumor becomes unresectable [12]. This opens up the prospects of alternative medicines and natural products or their secondary metabolites, have been demonstrated to have hepatoprotective and anticancer benefits [6, 7]. DEN is a strong hepatocarcinogenic substance that causes disturbances in nucleic acid repair mechanisms and also generates reactive oxygen species (ROS) leading to oxidative stress [13]. As such in the present study, we utilized DEN to develop the rat model of HCC, and evaluated the hepatoprotective and antitumour properties of ADE. ADE effectively restored the normal liver architecture, oxidative marker status and cytokines balance (Fig. 6).

Following DEN, the characteristic histological features of liver, namely the hexagonal lobular structure with radially arranged hepatocytes, the central vein in the middle and peripheral portal triad were disorganized or lost. This could be attributed to the chain of events starting with oxidative stress, leading to membrane damage, inflammatory cell infiltration, cellular atypia and eventual transformation into HCC [14]. Administration of ADE not only inhibited the adverse effects of DEN but also helped in partial to complete restoration of histological features (Fig. 1). The reversal of the toxic effects of DEN by ADE is probably due to reduction or complete inhibition of the oxidative stress and improvement in the antioxidant status (Fig. 2). This is supported by the results observed in the present study where, the antioxidant enzymes namely SOD, GR, GPx and CAT were increased and LPO was reduced. Restoration of the antioxidant system and improvement in both liver structure and function by ajwa dates has been reported in earlier studies in response to different hepatocarcinogens [5, 15]. Luteolin, a bio-active flavonoid present in fruits and vegetables are known to inhibit the oxidative stress and restore histopathological features following DEN toxicity [14]. Luteolin is present in ajwa dates [8], and this probably contributed to the reversal of oxidative stress and restoration of normal liver architecture in the present study.

In response to a hepatotoxic agent the liver enzymes namely ALT, AST, ALP, gamma glutamyl transpeptidase increase and are indicative of hepatic insufficiency [7, 16]. In the present study too, elevated levels of ALT, AST and ALP with DEN treatment were reduced following ADE (0.5 g/kg bw and 1.0 g/kg bw for 10 weeks) (Fig. 3). Similar to our findings, pre- and post-administration of date fruit extract to rats treated with dimethoate, a liver toxic agent had helped restoration of ALT and AST to near normal levels [17]. In yet another study [18], where carbon tetrachloride (CCl_4_) induced hepatotoxicity in rats were used, the elevated levels of the liver enzymes ALT, AST and ALP returned close to their baseline values upon treatment with date seed extracts (1 g/kg bw for 4 weeks).

The polyphenolic compounds comprising of phenolics, flavonoids, stilbenes and lignins present in various plants, fruits and vegetables are known to exert hepatoprotective effects [7]. The total phenolic content measured as from date palm flesh is approximately 290 mg/100 g and in date seeds it is 38.8 mg/g [8], expressed as gallic acid equivalent. Therefore, the observed hepatoprotective effect following ADE treatment in DEN-induced HCC model (Fig. 6) in the present study could probably be due to the high phenolic contents in the palm date flesh.

Cytokines are the pleiotropic hormones of the immune system that play a pivotal role in the initiation, maintenance and progression of tumors [19]. Neoplastic cells and tumor-associated macrophages (TAM) secrete IL-6, CSF-1, IL-10, TGF-β, TNF-α, IL-1α, angiogenic and lymphangiogenic growth factors that promote tumor development [20, 21]. In our study, DEN administration specifically increased the serum levels of proinflammatory cytokines, such as IL-1α, IL-1β, GM-CSF, and treatment with ADE effectively attenuated these cytokines (Fig. 4). Studies have shown that flavonoids have anti-tumor and anti-inflammatory properties and are capable of reducing the serum levels of Th1 cytokines [22]. The presence of major flavonoids such as apigenin, quercetin, and luteolin in ajwa could be one of the reason for reduced serum levels of proinflammatory cytokines (IL-1β, TNF-α and IL-6), and hepatoprotection observed in DEN-induced HCC in rats [23, 24]. Furthermore, lower levels of GM-CSF by itself effectively reduces the concentrations of other proinflammatory cytokines IL-1β and TNF-α [25].

In the present study, that the levels of both IL-2 and IL-12 were reduced significantly with DEN treatment but increased following ADE administration. Another pro-inflammatory cytokine IL-4, which is implicated in tumor promoting activities however, remained unaffected with DEN treatment compared to untreated normal. The cytokines IL-2 and IL-12 although considered to be pro-inflammatory, they negatively regulate tumor behavior [26, 27], and are currently in clinical use/trials [26]. Proanthocyanidins were reported to decrease the inflammation associated with 2,4-dinitrofluorobenzene and ultraviolet B UVB treatment in mice, by increasing the IL-12 concentration [28]. Proanthocyanidins are found in dietary fruits including ajwa dates and this could be attributed to the observed increase in the levels of IL-12 [7, 28].

Cancer is associated with inflammation and some of the inflammatory markers such as TNF-α, IL1, IL-6 become increased in HCC and after DEN administration [29–31], as both are associated with inflammation. In addition, cancer diagnosis or prognosis depends on identification of biomarkers. AFP is a commonly used marker in HCC and its serum [32, 33]. AFP and its receptor have been implicated in HBV mediated malignant transformation of hepatocytes and also in their metastasis via PI3K/AKT-mediated signaling [33]. In the present study we observed that both AFP and IL-6 gene expression were downregulated in response to ADE treatment (Fig. 5). Administration of luteolin (20 μg/kg bw, intraperitoneally) in mice decreased the plasma levels of AFP that was initially increased after DEN treatment [22]. Similarly, luteolin administration (30 mg/kg bw, orally) decreased the gene expression levels of IL-6 in DEN and ethanol-mediated hepatic inflammation in mice [34]. Ajwa dates or its constituents namely luteolin thus appears to have beneficial properties against HCC.

## Conclusions

ADE demonstrated significant beneficial effects against DEN induced HCC in rats. The anti-inflammatory, hepatoprotective and anticancer properties observed in the present study could be due to the presence of flavonoids such as luteolin, apigenin, quercetin and proanthocyanidins in dates which are rich in polyphenolic compounds. As such dates and or its constituents can be taken together with conventional chemotherapeutics, for synergistic benefits against HCC.

## Abbreviations

ADE: Aqueous Extract of ajwa Dates
AFP: Alpha-feto protein
AHU: Animal holding unit
ALP: Alkaline phosphatase
ALT: Alanine aminotransferase
AST: Aspartate aminotransferase
bw: Body weight
CAT: Catalase
DEN: Diethylnitrosamine
g: Gram
GM-CSF: Granulocyte-macrophage colony-stimulating factor
GPx: Glutathione peroxidase
GR: Glutathione reductase
HCC: Hepatocellular carcinoma
hrs: Hours
IL-1α: Interleukin-1alfa
KFMRC: King fahd medical research center
kg: Kilogram
KSA: Kingdom of Saudi Arabia
LPO: Lipid peroxidation
MDA: Malondialdehyde
mg: Milligram
min: Minute
nm: Nanometer
no: Number
PCR: Polymerase chain reaction
RNA: Ribonucleic acid
rpm: Rotation per minute
SEM: Standard error mean
SOD: Superoxide dismutase
